# Laboratory testing of extravascular body fluids: National recommendations on behalf of the Croatian Society of Medical Biochemistry and Laboratory Medicine. Part II – Synovial fluid

**DOI:** 10.11613/BM.2020.030501

**Published:** 2020-08-05

**Authors:** Anja Jokic, Lara Milevoj Kopcinovic, Jelena Culej, Irena Kocijan, Marija Bozovic

**Affiliations:** 1 Croatian Society of Medical Biochemistry and Laboratory Medicine, Working group for extravascular body fluid samples; 2 Department of Medical Biochemistry, Haematology and Coagulation with Cytology, University Hospital for Infectious Diseases “Dr. Fran Mihaljević”, Zagreb, Croatia; 3 Department of Clinical Chemistry, Sestre milosrdnice University Hospital Center, Zagreb, Croatia; 4 Medical Biochemistry Laboratory, General hospital Varaždin, Varaždin, Croatia

**Keywords:** recommendations, synovial fluid, crystal analysis, osteoarthritis, gout

## Abstract

Joint diseases are conditions with an often progressive and generally painful nature affecting the patient’s quality of life and, in some cases, requiring a prompt diagnosis in order to start the treatment urgently. Synovial fluid (SF) laboratory testing is an important part of a diagnostic evaluation of patients with joint diseases. Laboratory testing of SF can provide valuable information in establishing the diagnosis, be a part of a patient’s follow-up and treatment with the purpose of improving the patient’s health and quality of life. Synovial fluid laboratory testing is rarely performed in Croatian medical biochemistry laboratories. Consequently, procedures for SF laboratory testing are poorly harmonized. This document is the second in the series of recommendations prepared by the members of the Working group for extravascular body fluid samples of the Croatian Society of Medical Biochemistry and Laboratory Medicine. It addresses preanalytical, analytical, and postanalytical issues and the clinical significance of tests used in SF laboratory testing with the aim of improving the value of SF laboratory testing in the diagnosis of joint diseases and assisting in the achievement of national harmonization. It is intended for laboratory professionals and all medical personnel involved in synovial fluid collection and testing.

## Introduction

Chronic rheumatic conditions comprise more than 150 different diseases (including joint disease) with an often progressive and generally painful nature. They are among the leading causes of morbidity and disability worldwide and represent an enormous burden on healthcare systems (*1*).

Joint disease is often accompanied by alterations in the composition and volume of synovial fluid (SF). Laboratory testing of the SF can contribute to the differential diagnosis of rheumatic conditions accompanied with joint effusions. Indications for SF laboratory testing include inflamed joints with a known or unknown aetiology, suspected acute prosthetic joint infection, as well as infection identification by microbiological analyses and diagnosing crystal-induced arthritis, *etc.* Distinguishing amongst non-inflammatory and inflammatory joint effusions is considered the most important clinical application of SF laboratory testing. Furthermore, the clinical significance of SF laboratory testing has been established in acute arthritis, especially in the diagnosis of septic and crystal arthritis, as well as intercritical gout. Laboratory testing of SF can provide valuable information in establishing the diagnosis of a rheumatic condition, be a part of patient’s follow-up and treatment with the purpose of improving the patient’s health and quality of life (*2*-*5*).

The Working group for extravascular body fluid samples (WG EBFS) of the Croatian Society of Medical Biochemistry and Laboratory Medicine (CSMBLM) conducted an extensive survey aimed at mapping critical areas in the preanalytical, analytical, and postanalytical phases of extravascular body fluids analysis in Croatia. The main results demonstrated that SF laboratory testing is rarely performed in Croatian medical biochemistry laboratories, and since the procedures used in other investigated extravascular body fluid testing are not harmonized, the same level of harmonization might be expected in SF laboratory testing (*6*). These results, combined with a thorough review and critical assessment of all available scientific evidence, were used to design this document.

This recommendation is the second in the series of recommendations prepared by the members of the WG EBFS of the CSMBLM (*7*). It addresses the preanalytical, analytical, and postanalytical issues and clinical significance of tests used in SF analysis with the aim of improving the value of SF laboratory testing in the diagnosis of joint diseases and assisting in the achievement of national harmonization of SF testing. The ultimate goal is to improve patient safety and healthcare outcomes.

This document is intended for laboratory professionals and all medical personnel involved in the synovial fluid collection and analysis. It is organized in sections referring to the preanalytical, analytical and postanalytical phases of SF laboratory testing. Specific recommendations are presented in a box at the beginning of each section, followed by explanations and data derived from relevant literature. Similar to the first WG EBFS’s recommendation dedicated to serous fluids laboratory testing, cytological and microbiological testing are beyond the scope of this document since they are not performed in Croatian medical biochemistry laboratories (*7*).

## 1. Synovial fluid

Movable (diarthrodial) joints are enclosed in synovial cavities called synovia, which are filled with a viscous synovial fluid, produced by the ultrafiltration of plasma through the synovial membrane (and its adjacent capillaries) with the incorporation of hyaluronic acid secreted by synoviocytes. Synovial fluid glucose and uric acid concentrations resemble that of plasma, while its protein concentration is much lower compared to plasma protein concentrations. Synovial fluid also contains high amounts of locally synthesized hyaluronic acid, which contributes to the fluid’s viscosity. The main functions of SF are to lessen the friction between joints facilitating their free movement, to provide nutrients to the metabolically active and vascular-deficient cartilage, and to remove waste metabolites.

Physiologically, only small volume of SF (up to about 3.5 mL) is present in the synovium. Conditions including infection, inflammation, metabolic disorders, trauma, advanced age, *etc*., are associated with the accumulation of SF in the joint cavity. Laboratory testing of SF effusions is an additional and helpful tool for the diagnosis and classification of disorders affecting the articular membrane (*i.e.* arthritides). The SF analyses with the highest clinical value are SF crystal detection in crystal-associated synovitis (gout and/or pseudogout), and SF total and differential cell count in the confirmation of inflammatory (septic) arthropathies. Other biochemical analyses are neither specific nor sensitive but might provide useful additional information and narrow down the differential diagnosis of conditions affecting the joint. Combining the results of SF laboratory testing with the patient’s medical history and physical examination, arthritic disorders can be classified into four groups: non-inflammatory, inflammatory, septic and haemorrhagic (*2*, *8*-*12*). Table 1 summarizes SF laboratory testing results associated with previously mentioned joint conditions (*2*, *8*, *10*-*13*).

**Table 1:** Classification of joint effusions according to SF laboratory testing

**Parameter**	**Normal SF**	**Non-inflammatory SF**	**Inflammatory SF**	**Septic SF**	**Haemorrhagic SF**
Volume, mL	< 3.5	> 3.5	> 3.5	> 3.5	> 3.5
Appearance (colour, clarity)	Colourless – straw; clear	Lightly yellowish – straw; clear, slightly cloudy	Yellow-white, grey; cloudy, turbid, milky	White, grey, yellow-green; turbid, purulent	Red-brown, xanthochromic; turbid
Leukocyte count, x10^6^/L	< 200	20–2000	2000–50,000	> 50,000	Equal to blood
Neutrophils, %	< 25	< 25	> 50	> 75 (> 90)	Equal to blood
Serum-synovial fluid glucose difference (mmol/L)	≤ 0.6	< 1.1	> 1.1 (to 4.4)	> 2.2 (to 5.6)	< 1.1
Crystals	None	Occasionally calcium-pyrophosphate and hydroxyapatite	Needle-shaped monosodium urate monohydrate (gout); rhomboid calcium pyrophosphate (pseudogout)	None	None
Possible disorders	None	Osteoarthritis, traumatic arthritis, osteonecrosis	Rheumatoid arthritis, septic arthritis, gout, pseudogout, SLE	Bacterial, fungal, mycobacterial infection	Trauma, haemophilia, anticoagulation therapy, tumour, joint prosthesis
SF – synovial fluid. Glucose difference – the difference of glucose concentrations between serum and SF, when serum samples are collected simultaneously. SLE – systemic lupus erythematosus. Adapted from (*2*, *8*, *10*-*13*).					

## 2. Preanalytical phase

2.1 **Patient preparation**

**Table:** 

No specific patient preparation procedure is needed before sample collection (arthrocentesis). If glucose is to be measured in the SF sample, the patient should be fasting at least 6 hours prior to the SF collection (*11*).

Since SF constituents mirror plasma concentrations, the recommended fasting period is necessary to equilibrate plasma and joint fluid concentrations in order to obtain reliable results of the SF analysis (*8*, *11*).

2.2 **Test request form and test ordering**

**Table:** 

The test request form for synovial fluid laboratory testing should adhere to accreditation and good laboratory practice requirements. It should contain the patient’s name, surname, gender, date of birth and a unique identifier (*e.g.* health insurance number), collection date and time, collection location (hospital ward), identification of the ordering physician and their contact details, identification of the clinical staff that performed the collection. The diagnosis and tests requested should be clearly indicated. If the sample is to be analysed as urgent, this should be clearly indicated on the request (*14*).

2.3 **Patient and sample identification**

**Table:** 

Samples should be labelled in the presence of the patient, with at least two unique identifiers (name and date of birth, preferably), location (ward), date and time of collection and anatomic site of collection (*e.g.* left knee, right elbow) (*15*, *16*).

Improperly identified (or unlabelled) sample containers should not be accepted for analysis (*15*). Sample rejection should be documented by the laboratory and stated on the patient’s report.

2.4 **Synovial fluid collection and handling**

**Table:** 

Appropriate collection containers and sample handling procedures (transport and processing) should be directed by the test ordered and should reflect appropriate procedures used for the validated (standard) sample type (*11*). The recommended synovial fluid sample volume *per* container is 3-5 mL. Synovial fluid samples for biochemical analyses should be collected in non-anticoagulated tubes (*e.g.* red top). Plain tubes without additives (*e.g.* white top) are also acceptable. Synovial fluid for cell count, differential cell count, viscosity, and crystal analysis should be preferably collected in tubes containing liquid ethylenediaminetetraacetic acid (EDTA) (lavender top). Alternatively, plain tubes without additives are also acceptable (*15*-*17*). Synovial fluid samples should be transported to the laboratory at room temperature immediately after collection (*i.e.* within one hour) in order to prevent cell degradation and alteration of biochemical components. Refrigerated SF samples are not suitable for the crystal analysis since cooling might induce *in vitro* precipitation of crystals. When necessary for interpretation purposes, a serum sample should be collected simultaneously to the arthrocentesis procedure (*8*, *10*, *11*, *15*).

Synovial fluid is collected by arthrocentesis (*i.e.* aseptic needle aspiration from a joint) which is performed for diagnostic and therapeutic purposes (*11*). This collection process is performed outside the laboratory and is coordinated as well as executed by trained clinicians. Efforts should be undertaken to standardize the SF collection process since improper collection techniques could seriously impact SF test results (*8*, *15*, *18*). Aspirations not performed according to standardized procedures increase the risk of causing blood vessel damage, which can result in a haemolysed SF sample (*19*). Furthermore, the clinician performing the SF collection should be made aware if the patient is taking any medications. Available literature data suggests that arthrocentesis might be safely performed in patients receiving warfarin and direct oral anticoagulants without altering their anticoagulation regimen (*20*, *21*).

Immediately after collection, an aliquot of the SF collected should be transferred to an appropriate container (tube). If anticoagulant containing tubes are used, they should be mixed gently according to the manufacturer’s instructions to ensure mixing of anticoagulants and SF sample. These steps should be done at the site of the collection before transportation to the laboratory (*15*).

The Clinical Laboratory Standards Institute (CLSI) recommends the following collection order: the first aliquot of the SF sample should be intended for biochemical analysis, the second aliquot for microscopic examinations (cell count, differentials, and crystal identification), while the third should be intended for microbiological analyses (*2*, *8*, *15*). The sample should be observed for clotting in case of non-anticoagulated tubes (normal SF does not clot, due to lack of fibrinogen). After clotting completion and centrifugation for 10 minutes, at 1000-3000 rounds *per* minute (rpm), biochemical testing is performed from the supernatant (*9*, *10*, *15*, *19*).

Tubes containing oxalate, lithium heparin or lyophilised EDTA are not recommended, because they may form crystalline-resembling formations, which can be mistaken for monosodium urate (MSU) or calcium oxalate crystals, causing false positive results (*2*, *10*, *15*, *19*).

The volume of synovial effusion varies depending on the condition and the joint that is affected. Often during the collection procedure, it is not possible to obtain a valid amount of SF as recommended. Volumes of synovial fluid less than 1 mL, collected in tubes containing liquid anticoagulants, can result in the destruction of cell components. However, low volumes of SF collected in plain tubes should not be rejected by the laboratory because microscopic analysis (*e.g.* cell count, differentials) and crystals diagnostics are feasible from a few drops of the SF sample. If a recommended sample volume is not available, the clinician should prioritize the test requested according to the suspected diagnosis in collaboration with the laboratory (which acknowledges the sufficiency of the sample volume for the requested tests) (*7*, *19*).

Synovial fluid samples delivered to the laboratory for analysis in large syringes are generally not acceptable, especially regarding synovial fluid sample clotting (*15*). The practice of delivering SF samples to the laboratory in aspiration syringes (especially with needles) represents a potential biohazard and should be avoided. However, considering the peculiarities of this sample type and its collection procedure, the SF should not be rejected by the laboratory solely based on inadequate container choice or non-compliant timeframe for sample delivery to the laboratory. Sample rejection criteria should be instituted and followed; SF sample rejection should be documented by the laboratory and stated in the patient’s report (*8*, *15*, *19*).

2.5 **Assessing sample quality**

**Table:** 

The quality of the sample should be assessed by visual inspection before analysis to avoid instrument failures and/or measurement errors. Clotted samples affect cell count accuracy and should not be considered suitable for these particular analyses. Inadequate sample quality should be documented in the test report (*11*, *15*).

Due to its high molecular weight, fibrinogen normally cannot pass through the synovial membrane. The presence of fibrinogen in the SF will cause sample clotting and may indicate a traumatic tap or a pathological disorder. To prevent sample clotting, an aliquot of the sample should be transferred in tubes containing liquid EDTA (*8*, *11*).

## 3. Method validation and quality control in synovial fluid laboratory testing

**Table:** 

A peruse of the manufacturer’s performance specifications for methods used in SF analysis is a precondition prior to the determination of specific biochemical parameters in the SF. If performance specifications for SF analysis are not provided in the manufacturer’s product insert, analytical method validation has to be undertaken, accompanied with appropriate documentation.
Recommendations for method validation as well as quality control and proficiency testing in the EBF analysis are available in the first recommendation of the WG EBFS and should be applied in the SF analysis (*7*, *15*).

## 4. Analytical phase

**Figure 1:**
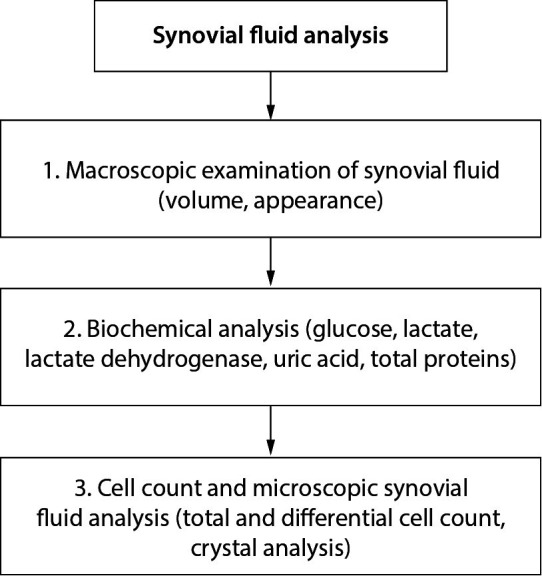
Algorithm for synovial fluid laboratory analysis.

Laboratory analysis of synovial fluid comprises of macroscopic examination (volume, appearance), the determination of specific biochemical analyses, and microscopic examination (Figure 1).

### 4.1 Macroscopic examination

4.1.1 **Synovial fluid volume**

**Table:** 

The total volume of synovial fluid collected should be recorded by the clinician immediately after arthrocentesis on the test request form and should later be stated in the laboratory test report (*10*, *19*, *22*).

The accumulation of more than 3.5 mL of SF can be considered an abnormal volume, indicating an intra-articular process of different ethology (*e.g.* inflammatory). However, small SF volumes do not exclude a joint condition. The presence of particles like inclusion bodies or fibrin can lead to difficulties in obtaining an SF sample, which might result in a falsely low volume collected (*10*, *11*, *19*, *22*).

4.1.2 **Synovial fluid appearance**

**Table:** 

Synovial fluid appearance (colour and clarity) should be determined visually, upon sample receipt and before centrifugation (*11*). It should be stated on the test report. Although not specific, SF appearance might provide useful diagnostic information concerning joint inflammation and presence of haemarthrosis. Normal SF is colourless (yellowish) and clear (*5*, *8*, *19*).

Synovial fluid appearance is an essential part of SF laboratory analysis. Synovial fluid appearance may indicate various disorders as well as a traumatic tap. If the SF is red-brownish and the colour is unevenly distributed, or a bloody stripe is noticed in the sample, a traumatic tap should be suspected (*2*, *8*, *10*, *11*, *19*). The clarity of SF might be modified by the presence of either increased numbers of white blood cells, or erythrocytes, synoviocytes, a multitude of MSU crystals, fibrin, cellular debris, lipids (*e.g.* in fat necrosis), chyle droplets, *etc.* (*8*, *11*, *19*). White free-floating rice-like aggregates, called rice bodies, may be present in SF samples. These particles are composed of collagen covered with fibrinous tissue and are frequently found in rheumatoid arthritis as a result of synovium degeneration. Ochronotic shards may be also seen in SF samples. These appear like ground pepper particles and represent pigmented pieces of cartilage originating from ochronotic arthropathy or metal and plastic joint prosthesis (*8*, *11*, *19*). Macroscopic characteristics of SF fluid in health and different joint disorders are presented in Table 1.

4.1.3 **Synovial fluid viscosity**

**Table:** 

The determination of synovial fluid viscosity is of low clinical value and should not be performed routinely (*8*).

Regardless of its limited clinical significance, SF viscosity is still assessed because of its cost-effectiveness and simplicity. The degree of viscosity of the SF is assessed by the string test. The string test can be determined directly by the clinician while transferring the SF sample from the collection syringe to the appropriate container. Alternatively, the string test can be performed before centrifugation by laboratory staff placing one drop of synovial fluid between two gloved fingers, a thumb, and an index finger. By moving fingers slowly in the opposite direction, a 5-centimetre-long string should be formed between them before breaking. If the string formed is < 3 centimetres long (low viscosity), it might suggest an ongoing inflammatory process in the joint. A string longer than 6 centimetres is found in cases of septic arthritis. Although accurate measurements of SF viscosity can be performed using s viscometer, this is rarely performed due to the low clinical value of this parameter (*11*, *19*).

Synovial fluid viscosity is physiologically very high and depends on the amount of polymerized hyaluronate present. Normal SF has a hyaluronate concentration of 3.0–3.5 g/L. These concentrations decrease physiologically after the age of 50, and at about the age of 80, their values are approximately 2 g/L. Furthermore, hyaluronate can be depolymerized by the hyaluronidase present in neutrophils and bacteria during inflammatory conditions of the joint. Additionally, some conditions prevent the synthesis of hyaluronate by synoviocytes. The result is a decreased (low) viscosity of the SF indicating the presence of an inflammatory process in the joint. The protein SF content, as well as the protein type, cells, temperature, and enzymes, have an impact on the SF viscosity (*8*, *10*, *11*, *18*, *19*, *22*, *23*).

4.1.4 **Synovial fluid mucin test**

**Table:** 

Although the SF mucin (also called the Rope’s) test is an indirect measure of SF viscosity, it is considered obsolete and should not be performed routinely (*8*).

The mucin clot test is a qualitative test that estimates the degree of polymerization of the hyaluronic acid-protein complex (mucin) responsible for SF viscosity. It provides little diagnostic information, but it is still used by some laboratories because of its simplicity. Mucin test can be performed to differentiate the synovial fluid from other fluids of uncertain origin (*8*, *11*, *19*).

4.2 **Biochemical synovial fluid analysis**

**Table:** 

Biochemical and microscopic analysis of SF samples should be performed immediately upon sample receipt (*i.e.* within 2 hours after collection) to avoid unreliable results. The first tube collected without additive should be inspected for clotting and then centrifuged to remove cellular and other components. The supernatant is used for chemical analyses. Very viscous synovial fluid samples should be pre-treated with a hyaluronidase solution to reduce sample viscosity, and then analysed. Biochemical analyses of the SF considered clinically useful are described below (*10*, *11*, *15*, *23*, *24*).

Synovial fluid has a similar chemical composition to plasma, although the concentrations of analytes found in normal SF are mostly lower compared to those found in the blood. Higher analyte concentrations are often related to intra-articular alterations and pathologic conditions.

Delayed laboratory analysis of SF samples might cause a reduction of the leukocyte number present in the sample; which is more pronounced in samples with higher leukocyte counts. Furthermore, prolonged sample storage (at room temperature or refrigerated) after collection might cause the artefactual formation and/or dissolution of crystals and the alteration of their optical characteristics. Thus, delayed analysis of SF samples could lead to the misdiagnosis of the underlying joint condition (*10*, *23*).

Different protocols for hyaluronidase solution preparation are available (*e.g.* 25 mg hyaluronidase and 1 mL of SF are incubated at 37ºC for 5 minutes, or 0.5 mg lyophilized hyaluronidase powder and 1 mL of SF incubate 15 minutes at room temperature) (*19*, *25*). A laboratory can also prepare plain microcentrifuge tubes with added hyaluronidase solution in advance and store them in a refrigerator or freezer prior to analysis. It is important in this course of action not to forget to calculate the dilution effect of hyaluronidase solution. Each laboratory should select the most suitable protocol for a routine application.

4.2.1 **Synovial fluid glucose**

**Table:** 

Synovial fluid glucose should be interpreted according to simultaneous glucose concentrations measured in the serum. Standard (serum) procedures should be applied to synovial fluid glucose measurement. Glucose concentrations in SF should be determined within one-hour form collection in order to prevent erroneously low glucose concentrations due to the glycolytic activity of leukocytes in the sample (*8*, *10*, *11*).

A simultaneous analysis of glucose concentrations in the serum and SF enables more reliable interpretation of glucose concentrations in the SF sample. In the fasting state glucose concentrations in the serum and SF are equivalent and the serum-synovial fluid glucose difference is ≤ 0.6 mmol/L. Conversely, in non-fasting patients, the serum-synovial fluid difference is > 0.6 mmol/L. Non-inflammatory and haemorrhagic conditions affecting the joint are characterized by a serum-synovial fluid glucose difference of < 1.1 mmol/L (Table 1). In inflammatory, crystal-induced and infectious joint conditions the serum-synovial fluid glucose difference is up to 2.2 mmol/L, 4.4 mmol/L, and 1.1–5.6 mmol/L, respectively. According to the Croatian Chamber of Medical Biochemistry (CCMB), the reference range for glucose in SF samples is 3.3–5.3 mmol/L (*8*, *11*, *15*, *26*).

4.2.2 **Synovial fluid lactate**

**Table:** 

The value of lactate measurement in SF is uncertain. Consequently, it should not be measured routinely. Synovial fluid lactate might be measured in case of suspected bacterial (septic) arthritis (*8*, *11*, *15*).

The measurement of lactate concentrations in SF is a helpful tool in the diagnosis of septic arthritis, especially in cases of negative bacteriologic culture results. The reference range for lactate concentrations in SF samples is 1.0–1.8 mmol/L. However, lactate concentrations increase with an increasing severity of inflammation, reaching concentrations as high as 13.5 mmol/L in culture positive SF samples. Lactate concentrations greater than 9.0 mmol/L strongly support the diagnosis of bacterial arthritis indicating the need for immediate treatment (*8*, *11*, *15*, *26*).

4.2.3 **Synovial fluid uric acid**

**Table:** 

The determination of uric acid in SF samples should be performed in cases of suspected gout without increased plasma uric acid concentrations, without urate crystals present microscopically or in laboratories without the necessary equipment for MSU crystal analysis (*8*, *11*, *15*).

The diagnosis of gout is established by the presence of patient’s symptoms and by measuring uric acid in plasma. The diagnosis is confirmed by the finding of MSU crystals in the SF. The determination of uric acid in SF samples is a helpful additional tool in gout diagnosis and should be measured using standard laboratory methods. Since SF uric acid concentrations are equivalent to those found in the, they are generally monitored by serum measurements (*8*, *11*, *15*).

4.2.4 **Synovial fluid total proteins**

**Table:** 

Synovial fluid proteins should be routinely measured in SF samples with standard (serum) procedures. The reference range of total proteins in SF samples is 11–22 g/L. Higher total protein concentrations are a nonspecific indicator of the presence of inflammatory joint disorders and are of little value in the differentiation of joint disorders or in guiding treatment (*11*, *25*-*27*).

Although all plasma proteins can be found in SF, high-molecular-weight proteins (*e.g.* α_2_-macroglobulin, β_2_-macroglobulin, and fibrinogen) are present only in very low concentrations or completely absent. Total protein concentrations in normal SF are about 1/3 of the protein concentration in plasma. The increased protein concentrations are caused by increased permeability of the synovial membrane or by increased synthesis in the joint cavity. Albumin represents the main protein fraction in SF with concentrations of approximately 12 g/L. The presence of ankylosing spondylitis, arthritis, arthropathies that appear as secondary consequences of Crohn disease, gout, psoriasis, ulcerative colitis, *etc.*, are accompanied by high total SF protein concentrations (*8*, *10*, *11*, *26*, *27*).

4.2.5 **Synovial fluid rheumatoid factor**

**Table:** 

Synovial fluid rheumatoid factor (RF) should not be routinely determined as a part of SF analysis. It might be determined as a confirmatory analysis in cases of rheumatoid arthritis (RA) that are not definitively diagnosed by standard (serum) analyses (*10*, *28*-*30*).

Synovial fluid RF can be found in approximately 60% of patients with RA in slightly lower concentrations than those found in serum (≤ 14 IU/mL). In general, synovial fluid RF determination in RA is not considered to be diagnostically helpful because positive RF may simply reflect serum concentrations and might derive from other chronic inflammatory conditions (*19*, *28*).

4.2.6 **Synovial fluid C - reactive protein**

**Table:** 

Synovial fluid C-reactive protein (CRP) should not be routinely measured to assess inflammation in the joint. However, SF CRP has demonstrated high sensitivity and specificity for the diagnosis of periprosthetic joint infection (PJI) and might be measured in patients with previous joint replacement and high clinical suspicion of PJI (*10*, *31*).

Nowadays, along with the CRP, human alpha-defensin 1-3 (AD) can be used for the diagnosis of PJI. Alpha-defensin has shown high diagnostic sensitivity and specificity for suspected PJI, especially after total knee and hip arthroplasty. The usefulness of AD has been demonstrated in ruling out PJI and it could be used as a confirmatory test for PJI (*32*-*35*).

4.2.7 **Synovial fluid lactate dehydrogenase**

**Table:** 

The activity of lactate dehydrogenase (LD) in SF samples should be measured as an indicator of the inflammation level present in the joint. The reference range of LD in SF is < 280 U/L (*11*, *26*).

Lactate dehydrogenase is the enzyme most frequently determined in SF samples. In normal SF, LD activity is lower compared to LD plasma activity. However, LD activities might be higher in SF samples, while normal activities are present in plasma. Lactate dehydrogenase activities of 400-700 U/L are related to moderate rheumatoid arthritis activity, while those exceeding 750 U/L indicate a high inflammatory activity. In general, high LD activities (*i.e.* above 280 U/L) are found in inflammatory effusions (*e.g.* gout, infectious arthritis, rheumatoid arthritis) (*11*, *15*, *26*).

### 4.3 Cell count and microscopic synovial fluid analysis

4.3.1 **Total and differential cell count in synovial fluid**

**Table:** 

Total and differential cell counts should be performed promptly upon the receipt of the SF sample (*i.e.* within one hour from the collection) using automated methods (analysers with a suitable mode for body fluid analysis). Alternatively, total and differential SF cell counts might be determined manually (by means of light microscopy). The normal SF total white blood cell (WBC) count is < 200 x10^6^/L. Manual differential WBC count should be performed in a stained cytocentrifuged preparation which allows the identification of cell types with diagnostic implications. The predominant cells found in SF are lymphocytes, monocytes, and macrophages, with a few neutrophils and synovial lining cells. The normal value of SF polymorphonuclear leukocytes (%PMN) is < 10%.
Although total WBC count and differentials have limited value in identifying specific joint conditions due to considerable intra-individual variations, it is accepted in clinical practice that the WBC count and %PMN moderately correlate with the degree of joint inflammation (*3*, *5*, *8*, *10*, *11*, *18*).

The white blood cell count and differential count are basic tools in the diagnosis and differentiation of various inflammatory and non-inflammatory conditions of the joint. Since the total WBC count gives overlapping results in the differentiation of joint disease categories in practice, the combination of WBC count and %PMN can be used to better discriminate non-inflammatory, inflammatory and infectious joint disorders. Differentials with %PMN > 80% are associated with bacterial arthritis and urate gout, while increased lymphocyte counts often occur in early stages of RA. The lack of harmonization of cell counting and differentiation presents a clear limitation in determining unique cut-offs for the diagnosis of various joint conditions. According to the American Rheumatism Association, the cut-offs for total WBC count and %PMN are as follows:

- normal SF - WBC < 200 x10^6^/L, PMN < 25%;
- non-inflammatory SF - WBC < 2000 x10^6^/L, PMN < 25%;
- inflammatory SF - WBC 2000–50,000 x10^6^/L, PMN > 50%;
- infectious SF - WBC > 50,000 x10^6^/L, PMN > 75% (Table 1) (*3*, *10*, *13*, *18*).

Available literature data suggest that anti-inflammatory drugs, especially nonsteroidal anti-inflammatory drugs (NSAIDs), in general, do not affect the total cell count in SF samples, although NSAIDs reduce the function of white blood cells (*e.g.* the production of proinflammatory cytokines) (*36*, *37*). However, in general, information concerning the intake of medications can be noted on the laboratory test request.

The presence of synovial lining cells is of no clinical significance. Abnormal numbers of cells or atypical cell types (*i.e.* plasma cells, eosinophils, lupus erythematosus cells, malignant) are indicative of various conditions affecting the joint (*8*, *11*). Synovial fluid samples with atypical cell types should be referred to cytological evaluation.

The total WBC with differentials should be performed within one hour of the collection to avoid cell destruction. However, accurate results can be obtained if SF samples are stored at 2-8°C in tubes containing EDTA additive (*38*, *39*). The undiluted SF sample should be thoroughly mixed prior to analysis. If significantly turbid, SF samples should be diluted with isotonic saline. Inflammatory SF fluids are prone to visible clotting, which might affect accurate cell count determination. Such SF samples should be analysed immediately or collected in tubes with appropriate additives. Erythrocytes have limited diagnostic value in SF samples. A high number of erythrocytes present in the SF sample derives either from haemorrhagic effusions or traumatic arthrocentesis. A high erythrocyte number might interfere with the WBC count and differential count. Consequently, erythrocytes should be selectively lysed by diluting the sample using hypotonic saline (0.3%) (*8*, *10*, *22*, *39*).

Manual cell counts in SF samples are performed using a haemocytometer (Neubauer or Fuchs- Rosenthal chamber). Due to SF sample’s high viscosity, the cells should be allowed to settle in the chamber for an additional period of time before counting. If necessary, sample viscosity should be reduced by dilution with hyaluronidase buffer enabling a more homogeneous cell distribution in the counting chamber (*8*, *10*).

Differential cell count should be performed by staining air-dried smears of cytocentrifuged SF samples according to May-Grünwald or Wright. Leukocyte morphology is then assessed under high power light microscope using oil immersion. Usually, mononuclear cells are predominant in non-inflammatory disorders while polynucleated cells prevail in inflammatory processes (*12*, *26*, *39*).

Although still considered the reference technique for counting and differentiation, optical microscopy is technically challenging, has a low throughput which results in longer turnaround times (TAT), is prone to substantial intra- and inter-observer variations and lacks standardization. Modern automated haematology analysers adapted for the SF counting analysis offer technical and clinical advantages. Compared to manual methods, automated counting is more reliable and practical, reduces TAT and enables longer cell stability. However, the limitations of automated cell counting methods should also be addressed, especially their inability to detect or correctly classify malignant cells and atypical leukocytes, their high imprecision (especially in samples with low cell counts), the interference of non-cellular particles (*e.g.* fat globules, cartilage fragments, crystals) which may cause pseudoleukocytosis or pseudoeosinophilia, and the presence of possible matrix effect which might affect proper sample aspiration. Therefore, unclear automated results, SF samples with low WBC count, suspected malignant samples, as well as suspected interferences should be verified by manual microscopy (*3*, *5*, *7*, *8*, *13*, *40*).

4.3.2 **Synovial fluid crystals**

**Table:** 

The presence of MSU is pathognomonic for urate arthritis (gout), while calcium pyrophosphate dihydrate (CPPD) crystals are associated with chondrocalcinosis (pseudogout). Synovial fluid crystal analysis should be performed using (direct or compensated) polarizing light microscopy (PLM). Slides should be prepared using cytocentrifuged SF samples to increase the sensitivity of crystal detection (*5*, *8*, *11*, *12*, *18*).

The identification of crystals by PLM is one of the most important analyses performed in SF samples. It is considered the gold standard for the definitive diagnosis of gout and pseudogout, especially in atypical cases. Compensated polarizing microscopy is the standard method for crystal identification. It allows the differentiation of synovial fluid crystals based on their birefringence. Synovial fluid crystals have the ability to refract polarized light in two dimensions at 90 degrees to each other and, depending on their molecular structure, to produce a characteristic colour under compensated polarized light. The compensator separates the light beam into slow- and fast-moving vibrations and produces a red background. Monosodium urate crystals are highly negatively birefringent and appear yellow when aligned with the slow vibrations, while CPPD is positively birefringent producing a blue colour (*10*, *41*, *42*).

Various types of crystals might be found in SF, two of the most frequent being MSU and CPPD. Other crystals (*e.g.* calcium hydroxyapatite, cholesterol, steroid crystals) have pathologic significance in the diagnosis of crystalline joint conditions (Table 2) (*5*, *8*, *10*, *11*, *43*).

**Table 2:** Synovial fluid crystals in joint conditions

**Crystal**	**Joint condition**	**Microscopic characteristics**
Monosodium urate	Urate arthritis (gout)	Fine, needle-like with strongly birefringent; extra- or intracellular (in leukocytes)
Calcium pyrophosphate	Chondrocalcinosis (pseudogout), degenerative arthritis, arthritis accompanying metabolic diseases	Rod-like, rhomboid, squared with a weak birefringence (best visualized by light microscopy)
Cholesterol	Chronic inflammatory conditions (RA)	Flat, rectangular plates, not birefringent
Hydroxyapatite	Apatite-associated arthropathies	Tiny, needle-like, not birefringent, present in leukocytes and visible only by electron microscopy
Corticosteroid	Months after intra-articular injection	Variable depending on the corticosteroid applied, similar to monosodium urate or calcium pyrophosphate
RA – rheumatoid arthritis. Adapted from (*8*, *10*, *11*, *22*, *24*).		

The temperature and variations in pH can affect crystal formation and solubility. Therefore, crystal analysis should be performed at room temperature as soon as possible after arthrocentesis (*8*, *10*, *22*). If MSU and CPPD crystal analysis are to be delayed or for educational purposes, the SF sample might be stored at room temperature (with or without sodium heparin or EDTA) up to 72 hours or at 4°C for eight weeks (*5*, *18*, *24*, *43*).

The cytocentrifugation prior to crystal analysis has several advantages: a) it concentrates SF components in a monolayer increasing sensitivity and recovery; b) the slides prepared might be retained for education, training and competency assessment; and c) the slides might be examined under microscope stained or unstained (*8*, *44*, *45*). Nevertheless, cytocentrifugation has several limitations: a) it requires more time to prepare the slide; b) it requires special equipment (cytocentrifuge); and c) in the presence of a large number of cells, the identification of crystals is more difficult. Alternatively, in the absence of a cytocentrifuge, slides for microscopic crystal analysis can be prepared using a conventional centrifuge, centrifuging samples for 10 minutes at 700 rpm (*8*, *46*).

Wet slides for SF crystal analysis are prepared from native samples. One drop of a thoroughly mixed SF sample is placed on a slide and covered with a coverslip. The sample should fill the area under the coverslip. The differentiation of SF crystals is difficult because their number might vary significantly; they are very similar, may be hidden in fibrin or cellular debris, and may be mistakenly identified as artefacts. Therefore, slides should be examined by experienced laboratory personnel. Slide examination should start with a low-power field screening procedure (100x magnification) followed by the identification of crystals using a high-power field (HPF) magnification (400x or higher). A crystal positive sample of the SF should contain at least two typical shapes (*e.g.* MSU needle-like shape) in each field of 10 randomly selected fields of view. Crystals description should include birefringence (strong, weak), shape (needle, rhomboid, square bipyramidal, *etc.*), cell location (intra-, extracellular) and quantity (number of crystals as *per* HPF) (*8*, *10*, *22*, *25*, *40*).

## 5. Postanalytical phase

**Table:** 

Laboratory test reports of SF analysis should include the type of fluid analysed, the measured value, as well as reference range and/or decision limits for each tested analyte in order to guide clinical interpretation and decision-making. Additionally, test reports should include a comment acknowledging the possible influence of sample matrix on the test’s accuracy and thus the need to interpret results in conjunction with clinical symptoms (*7*, *15*). If methods used in SF analysis have not been validated in the laboratory, a comment should be included on the test report. Furthermore, the laboratory should contact the ordering physician prior to issuing such laboratory test reports to explain potentially influencing factors. Laboratories are strongly encouraged to communicate and comment the results obtained by SF analysis with the ordering/responsible clinical personnel in order to aid diagnosis, better patient management or recommend further laboratory testing. Standardized interpretive comments should be included in the SF analysis test reports (*7*, *15*, *47*, *48*).

The results of SF biochemical analysis should be reported in the same units as those obtained in the serum/plasma. The values of simultaneous measurement of an analyte in the serum/plasma and synovial fluid (*e.g.* glucose) should be reported on the same test report (*15*, *48*). Decision limits and/or reference intervals for clinically useful analytes in SF laboratory testing are provided throughout this document and should be implemented on the test report (summarized in Table 3). Similarly, the recommended storage conditions might be applied, although laboratories should validate the stability of the analytes tested in SF samples in their own routine setting (*7*, *15*).

**Table 3:** Reference intervals and differential limits for clinically useful analytes

**Parameter**	**Normal SF**	**Inflammatory SF**	**Septic SF**
Glucose (mmol/L)	3.3–5.3	1.1–3.1	1.1–1.7
Lactate (mmol/L)	1.0–1.8	Up to 6.8	> 9.0
Uric acid (mmol/L)	Equal to blood	Equal to blood	Equal to blood
Total proteins (g/L)	11–22	> 40	30–60
RF	Negative	Positive/negative	Negative
LD (U/L)	< 280	> 280 (to 750)	> 300
WBC (x10^6/^L)	< 200	2000–50,000	50,000
%PMN	< 25	> 50	> 75
Monosodium urate crystals	Negative	Positive	Negative
Calcium pyrophosphate crystals	Negative	Positive	Negative
SF – synovial fluid. RF – rheumatoid factor. LD – lactate dehydrogenase. WBC – white blood cell count. PMN – polymorphonuclear lymphocytes. Adapted from (*2*, *8*, *10*, *12*, *13*, *15*, *19*, *26*).			

The test report should include interpretive comments addressing the preanalytical and analytical phases of SF analysis. If available, a laboratory information system should be used to generate standardized and clear comments (*7*). The practice of directly communicating and interpreting results with the ordering/responsible clinician should be encouraged.

## References

[1] World health organisation (WHO). Chronic diseases and health promotion. Available at: http://www.who.int/chp/topics/rheumatic/en/. Accessed April 6th 2018.

[2] Sholter DE, Russell AS. Synovial fluid analysis. Available at: https://www.uptodate.com/contents/synovial-fluid-analysis. Accessed December 11th 2017.

[3] Seghezzi M Buoro S Manenti B Mecca T Ferrari R Zappala G Optimization of cellular analysis of synovial fluids by optical microscopy and automated count using the Sysmex XN Body fluid mode. Clin Chim Acta. 2016;462:41–8. 10.1016/j.cca.2016.08.018 27581597

[4] Osmon DR Berbari EF Berendt AR Lew D Zimmerli W Steckelberg JM Executive summary: diagnosis and management of prosthetic joint infection: clinical practice guidelines by the Infectious Diseases Society of America. Clin Infect Dis. 2013;56:1–10. 10.1093/cid/cis966 23230301

[5] Courtney P Doherty M Joint aspiration and injection and synovial fluid analysis. Best Pract Res Clin Rheumatol. 2009;23:161–92. 10.1016/j.berh.2009.01.003 19393565

[6] Kopcinovic LM Vogrinc Z Kocijan I Culej J Aralica M Jokic A Laboratory testing of extravascular body fluids in Croatia: a survey of the Working group for extravascular body fluids of the Croatian Society of Medical Biochemistry and Laboratory Medicine. Biochem Med (Zagreb). 2016;26:395–407. 10.11613/BM.2016.042 27812307 PMC5082222

[7] Milevoj Kopcinovic L Culej J Jokic A Bozovic M Kocijan I Laboratory testing of extravascular body fluids: National recommendations on behalf of the Croatian Society of Medical Biochemistry and Laboratory Medicine. Part I – Serous fluids. Biochem Med (Zagreb). 2020;30:010502. 10.11613/BM.2020.010502 31839720 PMC6904973

[8] Swan A Amer H Dieppe P The value of synovial fluid assays in the diagnosis of joint disease: a literature survey. Ann Rheum Dis. 2002;61:493–8. 10.1136/ard.61.6.493 12006320 PMC1754135

[9] Brunzel NA, editor. Fundamentals of urine and body fluid analysis. 3rd ed. Elsevier Saunders: St. Louis, 2013.

[10] King Strasinger S, Schaub Di Lorenzo M, editors. Urinalysis and body fluids. 5th ed. F. A. Davis Company: Philadelphia, 2008.

[11] Mundt LA. Synovial fluid. In: Mundt LA, Shanahan K. eds. Graff’s textbook of urinalysis and body fluids. 2nd ed. Philadelphia: Lippincott Williams and Wilkins; 2011. p. 253-262.

[12] El-Gabalavy HS. Synovial fluid analyses, synovial biopsy, and synovial pathology. In: Firestein GS, Budd RC, Gabriel SE, McInnes IB, O’Dell JR, eds. Kelley’s textbook of rheumatology. GRAD: Elsevier Saunders, 2013. Available at: http://peds.stanford.edu/Rotations/red_team/pdfs/lab/Kelley-Synovial%20Fluid%20Analyses,%20Synovial%20Biopsy,%20and%20Synovial%20Pathology.pdf. Accessed March 15th 2016.

[13] Fleming C Russcher H Lindemans J de Jonge R Clinical relevance and contemporary methods for counting blood cells in body fluids suspected on inflammatory disease. Clin Chem Lab Med. 2015;53:1689–706. 10.1515/cclm-2014-1247 25879321

[14] Croatian Society for Medical Biochemistry. Standardi dobre stručne prakse. Sadržaj uputnice. Available at: http://www.hkmb.hr/povjerenstva/strucna-pitanja.html Accessed March 23rd 2016. (in Croatian)

[15] Clinical and Laboratory Standards Institute. Analysis of body fluids in clinical chemistry. Approved guideline. Document C49-A. CLSI, Wayne, PA, USA: 2007.

[16] Clinical and Laboratory Standards Institute (CLSI). Body fluid analysis for cellular composition: Approved guideline. CLSI document H56-A. CLSI, Wayne, USA, 2006.

[17] Vrkic N, editor. Zglobna tekućina. In: Nikolac Gabaj N, ed. Ekstravaskularni uzorci u laboratorijskoj medicini. Zagreb: Medicinska naklada; 2019. p.137-46. (in Croatian)

[18] Lorenzini S, Morozzi G, Genco RL, Massenti MF, Ruggeri M, Scandone L, Tabacco F. Linee guida per l’analisi del liquido sinoviale: proposta. RiMeL/IJLaM 2008;4:8-15.

[19] Thomas L. Synovial fluid. In: Thomas L, ed. Clinical Laboratory Diagnostics. 1st ed. Frankfurt am Main: TH-Books; 1998. p.1381-92.

[20] Ahmed I Gertner E Safety of arthrocentesis and joint injection in patients receiving anticoagulation at therapeutic levels. Am J Med. 2012;125:265–9. 10.1016/j.amjmed.2011.08.022 22340924

[21] Yui JC Preskill C Greenlund LS Arthrocentesis and joint injection in patients receiving direct oral anticoagulants. Mayo Clin Proc. 2017;92:1223–6. 10.1016/j.mayocp.2017.04.007 28778256

[22] Martínez-Castillo A Núñez C Cabiedes J Synovial fluid analysis. Reumatol Clin. 2010;6:316–21. 10.1016/j.reuma.2009.12.010 21794741

[23] Kerolus G Clayburne G Schumacher HR Jr Is it mandatory to examine synovial fluids promptly after arthrocentesis? Arthritis Rheum. 1989;32:271–8. 10.1002/anr.1780320308 2930602

[24] Gálvez J Saiz E Linares LF Climent A Marras C Pina MF Delayed examination of synovial fluid by ordinary and polarized light microscopy to detect and identify crystals. Ann Rheum Dis. 2002;61:444–7. 10.1136/ard.61.5.444 11959769 PMC1754078

[25] Li B Yang S Akkus O A customized raman system for point-of-care detection of arthropathic crystals in the synovial fluid. Analyst. 2014;139:823–30. 10.1039/c3an02062b 24419093 PMC3932814

[26] Croatian chamber of medical biochemists (CCMB). Harmonizacija specijalističkih i visokodiferentnih pretraga iz područja medicinske biokemije, laboratorijske imunologije i analitičke toksikologije. Available at: http://www.hkmb.hr/obavijesti/obavijesti-index.html. Accessed May 24th 2016. (in Croatian)

[27] Hui AY McCarty WJ Masuda K Firestein GS Sah RL A systems biology approach to synovial joint lubrication in health, injury, and disease. Wiley Interdiscip Rev Syst Biol Med. 2012;4:15–37. 10.1002/wsbm.157 21826801 PMC3593048

[28] Body fluid reference intervals and/or interpretive information for select analytes. Available at: https://aruplab.com/bodyfluids. Accessed July 5th 2018.

[29] Rheumatoid Arthritis – RA. Available at: https://arupconsult.com/content/rheumatoid-arthritis. Accessed July 5th 2018.

[30] Caspi D Anouk M Golan I Paran D Kaufman I Wiggler I Synovial fluid levels of anti–cyclic citrullinated peptide antibodies and IgA rheumatoid factor in rheumatoid arthritis, psoriatic arthritis, and osteoarthritis. Arthritis Rheum. 2006;55:53–6. 10.1002/art.21691 16463412

[31] Wang C Wang Q Li R Duan JY Wang CB Synovial fluid C-reactive protein as a diagnostic marker for periprosthetic joint infection: a systematic review and meta-analysis. Chin Med J. 2016;129(16):1987–93. 10.4103/0366-6999.187857 27503025 PMC4989431

[32] Salinas M Rosas J Iborra J Manero H Pascual E Comparison of manual and automated cell counts in EDTA preserved synovial fluids. Storage has little influence on the results. Ann Rheum Dis. 1997;56:622–6. 10.1136/ard.56.10.622 9389224 PMC1752268

[33] Drago L Toscano M Tacchini L Banfi G α-Defensin point-of-care test for diagnosis of prosthetic joint infections: neglected role of laboratory and clinical pathologists. Clin Chem Lab Med. 2017;56:19–24. 10.1515/cclm-2017-0041 28708567

[34] Bonanzinga T Ferrari MC Tanzi G Vandenbulcke F Zahar A Marcacci M The role of alpha defensin in prosthetic joint infection (PJI) diagnosis: a literature review. EFORT Open Rev. 2019;4:10–3. 10.1302/2058-5241.4.180029 30800475 PMC6362339

[35] Gehrke T Lausmann C Citak M Bonanzinga T Frommelt L Zahar A The Accuracy of the Alpha Defensin Lateral Flow Device for Diagnosis of Periprosthetic Joint Infection: Comparison with a Gold Standard. J Bone Joint Surg Am. 2018;100:42–8. 10.2106/JBJS.16.01522 29298259

[36] Gallelli L Galasso O Falcone D Southworth S Greco M Ventura V The effects of nonsteroidal anti-inflammatory drugs on clinical outcomes, synovial fluid cytokine concentration and signal transduction pathways in knee osteoarthritis. A randomized open label trial. Osteoarthritis Cartilage. 2013;21:1400–8. 10.1016/j.joca.2013.06.026 23973155

[37] Raza K Falciani F Curnow SJ Ross EJ Lee CY Akbar AN Early rheumatoid arthritis is characterized by a distinct and transient synovial fluid cytokine profile of T cell and stromal cell origin. Arthritis Res Ther. 2005;7:R784–95. 10.1186/ar1733 15987480 PMC1175027

[38] Pascual E Jovaní V Synovial fluid analysis. Best Pract Res Clin Rheumatol. 2005;19:371–86. 10.1016/j.berh.2005.01.004 15939364

[39] Dougados M Synovial fluid cell analysis. Baillieres Clin Rheumatol. 1996;10:519–34. 10.1016/S0950-3579(96)80047-1 8876957

[40] Robier C Neubauer M Quehenberger F Stettin M Rainer F Calcium pyrophosphate and monosodium urate crystals in synovial fluid as a cause of pseudoeosinophilia. Clin Chem Lab Med. 2011;49:1345–7. 10.1515/CCLM.2011.212 21627492

[41] Graf SW Buchbinder R Zochling J White SL The accuracy of methods for urate crystal detection in synovial fluid and the effect of sample handling: A systematic review. Clin Rheumatol. 2013;32:225–32. 10.1007/s10067-012-2107-0 23138881

[42] Zhang Y, Lee SYC, Zhang Y, Furst D, Fitzgerald F, Ozcan A. Wide-field imaging of birefringent synovial fluid crystals using lens-free polarized microscopy for gout diagnosis. Scientific Reports. 6:28793. Available at: https://www.nature.com/articles/srep28793. Accessed June 22nd 2019. https://doi.org/10.1038/srep28793 PMC4928089 27356625

[43] Pascual E Sivera F Andrés M Synovial fluid analysis for crystals. Curr Opin Rheumatol. 2011;23:161–9. 10.1097/BOR.0b013e328343e458 21285711

[44] Robier C Stettin M Quehenberger F Neubauer M Cytospin preparations are superior to common smears in the detection of monosodium urate crystals in low-cellular synovial fluids. Clin Rheumatol. 2014;33:1797–800. 10.1007/s10067-014-2619-x 24744156

[45] Robier C Quehenberger F Neubauer M Stettin M Rainer F The cytospin technique improves the detection of calcium pyrophosphate crystals in synovial fluid samples with a low leukocyte count. Rheumatol Int. 2014;34:773–6. 10.1007/s00296-013-2689-0 23388697

[46] Boumans D Hettema ME Vonkeman HE Maatman RG van de Laar MA The added value of synovial fluid centrifugation for monosodium urate and calcium pyrophosphate crystal detection. Clin Rheumatol. 2017;36:1599–605. 10.1007/s10067-017-3633-6 28424907

[47] Kilpatrick E. Best Practice when providing interpretative comments on laboratory medicine reports. Available at: http://acb.org.uk/docs/default-source/committees/scientific/guidelines/acb/best-practice-when-providing-interpretative-comments-for-laboratory-medicine---final.pdf?sfvrsn=2. Accessed June 4th 2019.

[48] Vasikaran S Sikaris K Kilpatrick E French J Badrick T Osypiw J Assuring the quality of interpretative comments in clinical chemistry. Clin Chem Lab Med. 2016;54:1901–11. 10.1515/cclm-2016-0709 27641826

